# Assessment of Long-Term Effects of Nanoparticles in a Microcarrier Cell Culture System

**DOI:** 10.1371/journal.pone.0056791

**Published:** 2013-02-14

**Authors:** Maria Mrakovcic, Markus Absenger, Regina Riedl, Claudia Smole, Eva Roblegg, Leopold F. Fröhlich, Eleonore Fröhlich

**Affiliations:** 1 Center for Medical Research, Medical University of Graz, Graz, Austria; 2 Institute for Medical Informatics, Statistics and Documentation, Medical University of Graz, Graz, Austria; 3 Institute of Pathology, Medical University of Graz, Graz, Austria; 4 Institute of Pharmaceutical Sciences, Department of Pharmaceutical Technology, Karl-Franzens-University of Graz, Graz, Austria; National Institute of Health (NIH), United States of America

## Abstract

Nano-sized materials could find multiple applications in medical diagnosis and therapy. One main concern is that engineered nanoparticles, similar to combustion-derived nanoparticles, may cause adverse effects on human health by accumulation of entire particles or their degradation products. Chronic cytotoxicity must therefore be evaluated. In order to perform chronic cytotoxicity testing of plain polystyrene nanoparticles on the endothelial cell line EAhy 926, we established a microcarrier cell culture system for anchorage-dependent cells (BioLevitator^TM^). Cells were cultured for four weeks and exposed to doses, which were not cytotoxic upon 24 hours of exposure. For comparison, these particles were also studied in regularly sub-cultured cells, a method that has traditionally been used to assess chronic cellular effects. Culturing on basal membrane coated microcarriers produced very high cell densities. Fluorescent particles were mainly localized in the lysosomes of the exposed cells. After four weeks of exposure, the number of cells exposed to 20 nm polystyrene particles decreased by 60% as compared to untreated controls. When tested in sub-cultured cells, the same particles decreased cell numbers to 80% of the untreated controls. Dose-dependent decreases in cell numbers were also noted after exposure of microcarrier cultured cells to 50 nm short multi-walled carbon nanotubes. Our findings support that necrosis, but not apoptosis, contributed to cell death of the exposed cells in the microcarrier culture system. In conclusion, the established microcarrier model appears to be more sensitive for the identification of cellular effects upon prolonged and repeated exposure to nanoparticles than traditional sub-culturing.

## Introduction

In nanotechnology, a nanoparticle (NP) is defined as a small object that behaves as a whole unit in terms of its transport and properties. NPs are natural, incidental or manufactured particles with one or more external dimensions that range from 1 to 100 nm [1], [2]. NPs are of great scientific interest as they bridge bulk materials and atomic or molecular structures. Properties of nanomaterials (NMs) change as their size approaches the nanoscale [3]. Because of quantum size and large surface area, NMs have unique properties compared with their larger counterparts. Even when made of inert elements (e.g. gold), NMs become highly (re)active or even catalytic at nanometer dimensions [4], mostly because of their high surface to volume ratio. Oberdörster et al. discovered that the toxic effect of NMs is influenced by several properties, such as size, surface charge, hydrophobicity, shape and contamination [5]. Size and surface characteristics of NPs are no constants, but vary according to the concentration of salts and proteins as well as to mechanical pre-treatment [6]. The danger of inhaling particulate matter (fume or smoke particles) has been recognized since ancient times, but it was not until the early 1990s when associations between particle inhalation and diseases of the respiratory or cardiovascular systems were uncovered [7]. At that time, researchers started to systematically study the effects of (natural) NPs on human health [8], [9], especially the association between NP size and its response in lung tissue [10]–[12]. However, due to their properties, engineered NMs are increasingly often used in consumer products. But the same advantageous size-dependent properties of NMs lead to the possibility of harmful size-dependent biological interactions [13]. Therefore, the need to assess the potential risk of NMs on human health is rapidly growing.

NPs can display acute cytotoxic action at the site of entry. Cells important in this regard are epithelial cells of the respective organ, and cells of the innate immune system. Upon exposure to NMs, such as carbon black (CB), carbon nanotubes (CNTs), or zinc oxide, cells may be acutely damaged and their functionality may be compromised [14]–[17]. Both, bio-persistent (e.g. CNTs) and bio-degradable (e.g. iron oxide) NPs may cause severe problems [2], [18]. In addition to acute toxic effects, chronic exposure may result in selective cytotoxicity affecting specific cell functions [19]. However, testing of chronic effects *in vitro* is rarely done for conventional substances. Drugs are usually metabolized, excreted and degraded within cells and cellular accumulation is not expected. Consequently, models to assess chronic toxicity have not been developed and chronic toxicity is usually studied in animals. Nevertheless, data suggest that some NMs are not sufficiently cleared from the organism [20], [21]. If an organism is exposed over a long period to low concentrations of NPs, the function of cells may be compromised. Most indications for organ damage by repeated exposure to NPs were obtained in animal studies. Repeated exposure to gold NPs and magnetic NPs caused not only accumulation and histopathological changes in various organs but also weight loss and marked alterations in blood count [22]–[24]. Therefore, the assessment of toxic effects is becoming of outmost importance.

In short-term cytotoxicity studies, cell lines are usually employed, but these generally cannot be studied much longer than 72 hours in conventional culture. Subsequently, the cells need medium change and/or the cultures are in the stationary state. To assess longer time-periods, cells have been sub-cultured and again exposed to the tested compound [21]. Other systems such as bioreactors have to be used when observations over longer time-periods are needed [25], [26]. Dependent on their growth characteristics (adherent or in suspension), cells in bioreactors are either dispersed in medium or cultured on scaffolds, matrices or microcarriers.

In microcarrier cell cultures, anchorage-dependent cells are grown on the surface of small spheres which are maintained in stirred suspension cultures. In comparison to conventional monolayer cell culture, this technology provides the advantage that high cell densities and higher yields of cellular products such as antibodies can be obtained. Main advantages of the microcarrier system are reduced costs and reduced risk of contamination, increased culture periods without sub-culturing [27] as well as the imitation of the *in vivo* situation due to a more physiologic environment. This technique is therefore a good choice where cells are used for the production of biologicals, cells, cell products, and viral vaccines. Other applications include studies of cell structure, function and differentiation, enzyme-free sub-cultivation, and implantation studies [28]–[30]. Several cell lines (e.g. MDCK, Vero cells, Cos-7, stem cells, HEK 293T) were described to grow and differentiate on microcarriers [31]–[34].

In this study, we describe a microcarrier cell culture system to monitor cellular effects of NPs for a period of four weeks. We used plain polystyrene particles (PPS) as model NPs, as they are not bio-degradable; thus, the effect of accumulation can be studied. To investigate the suitability of the microcarrier system for other NMs, multi-walled CNTs were also evaluated. Cytotoxicity was assessed in microcarrier culture as well as in repeatedly sub-cultured cells. Moreover, the intracellular localization and the mode of cell death were investigated.

## Materials and Methods

### Cell culture

All experiments were performed on the endothelial cell line EAhy 926 which was kindly provided by Dr. C.J. Edgell [35]. Cells were cultured in high glucose Dulbeco’s Modified Earls Medium (DMEM) supplemented with 10% fetal bovine serum (FBS), 2 mMF⋅L^−^ Glutamine and 1% penicillin/streptomycin (P/S) (PAA, Austria).

### Nanoparticles

Non-functionalized PPS ‘Nanosphere Size Standards’ 1% (w/v) 20 nm and 200 nm, red fluorescent PPS ‘Fluoro-Max Red Aqueous Fluorescent Particles’ R25 1% (w/v) 25 nm (Thermo Scientific, USA), and short (0.5–2 µm) carboxyl-functionalized >50 nm diameter CNTs (MWCNT >50 nm COOH) (CheapTubes Inc., Brattleboro, Vermont) were used. CNTs were synthesized by catalytic chemical vapour deposition, acid purified, and were functionalized through repeated reductions and extractions in concentrated acids. As indicated by the supplier, CNTs were of high purity (>95%) with low amount of contaminants (ash <1.5 wt%).

### Characterization of particles

Particle characterization was performed by dynamic light scattering with a Malvern Zetasizer 3000 HS. Size and surface charge were determined after sonification for 20 minutes in distilled water, and in cell culture medium (DMEM) with or without 10% FBS.

### Cytotoxicity screening

1.4−1.7×10^5^ cells per ml were seeded in 96-well plates (Corning Costar, The Netherlands) and were incubated overnight at 37 °C and 5% CO_2_ to allow cell attachment. For cytotoxicity screening on Global Eucaryotic Microcarrier GEM™ (Global Cell Solutions, Virginia, USA), 2×10^5^ cells per ml were seeded in 96-well plates (Corning Costar) coated with a 5% poly (2-hydroxyethyl methacrylate) (poly-HEMA) solution (Sigma, Austria) to block cell attachment onto the plate. Cultures were exposed to different concentrations of 20 nm and 200 nm PPS as well as CNTs for 4 and 24 hours. After treatment, the viability of the cells was assessed by a formazan bioreduction (MTS) assay (CellTiter 96*®* AQueous Non-Radioactive Cell Proliferation Assay, Promega, Germany) according to the manufacturer’s protocol. After two hours of incubation with the MTS-solution, the absorbance was measured on a SpectraMAX plus 384 (Molecular Devices, Austria) at 490 nm. Wells without cells but with the respective medium, in which the NPs were dissolved, were used as blank control. To investigate whether the NPs interfere with the assay, an interference control ( =  highest concentration of each NP without cells) was included. In addition, after exposure to CNT, cells were washed three times with pre-warmed phosphate buffered saline (PBS) (PAA) prior to adding staining solution.

### Mode of action of PPS in conventional cell culture

After exposure of cells to the PPS for 4, 8, and 24 hours, the integrity of the cell membrane was determined using the CytoTox-ONE^TM^Homogeneous Membrane Integrity Assay (Promega), based on the release of lactate dehydrogenase (LDH). The fluorescence was recorded with an excitation wavelength of 560 nm and an emission wavelength of 590 nm on a FLUOstar Optima (BMG Labtech, Germany). As positive control, the cells were treated with a lysis solution of equal amounts of Triton X-100 and 70% ethanol for 10 minutes at room temperature. Induction of apoptosis was assessed after treatment of cells under same conditions as for the LDH measurement by using the Caspase-Glo 3/7 Assay (Promega). Measurements were read on a Lumistar (BMG Labtech). Both assays were carried out according to manufacturer’s instructions.

### Assays on long-term effects in conventional cell culture

Cells were plated in 25 cm^2^ cell culture dishes in complete DMEM and were incubated at 37 °C and 5% CO_2_ to allow cell attachment. After 24 hours, the media were exchanged and NPs at a final concentration of 20 µg/ ml were added. Controls received no NPs. Cell numbers and cell viability were assessed at time-points when controls reached 100% confluence to avoid bias by growth inhibition. In parallel, assays on the membrane integrity and apoptosis were assessed as described above and the cells were sub-cloned in a 1∶10 ratio.

### Microcarrier cell culture

For the establishment of a three dimensional model, basal membrane coated GEM™ were used. Cells were incubated in specialized culture vessels (LeviTubes^TM^) in the bench-top bioreactor BioLevitator™ (Hamilton Company, Switzerland) at 37 °C and 5% CO_2_. Two pre-installed culturing protocols (for epithelial and endothelial cells) were compared with respect to cell proliferation. 3×10^6^ cells were seeded on a 50% bead slurry in medium with 10% FBS. After an overnight inoculation period, LeviTubes™ were filled with additional medium. 20 nm and 200 nm PPS, in concentrations where no acute toxicity was observed after 24 hours, were added to the medium. For untreated controls no NPs were added. In parallel, red fluorescent labelled PPS were used in order to identify the sub-cellular localization of the NPs.

In pilot experiments, we investigated the effects on cell growth, viability, as well as on the toxic action of 20 nm PPS by changing the medium every other day (as in conventional cultures), and once per week. Since no differences on any outcome were observed, the medium was changed once per week in all further experiments.

### Microscopical evaluation of endothelial cells in microcarrier culture

Cell attachment was recorded at regular intervals by staining the nuclei with 5 µg/ ml Hoechst 33342 staining solution (Invitrogen, Austria) for 15 minutes at room temperature. The viability of cells was determined by labelling different cell organelles (nucleus, endoplasmic reticulum and mitochondria). An aliquot of the microcarrier cultures was stained with Hoechst 33342, 1 µM ER-Tracker green and 200 mM MitoTracker DeepRed 633 (Invitrogen). After incubation for 20 minutes at 37 °C and 5% CO_2_, staining was documented on a LSM510 Meta confocal laser scanning microscope (Zeiss, Germany). Changes in cell number and viability were recorded weekly. GEM™ were dissolved in pre-warmed trypsin/EDTA (PAA). Detachment of the cells was observed under a CKX41 light microscope (Olympus, Japan). Cell number and viability was determined using a CasyONE*®* cell counter (Inovatis, Germany) [36].

### Mode of NP action in microcarrier cell cultures

Induction of apoptosis and/or necrosis of the long-term exposed cultures were assessed weekly and were compared to untreated controls. Mean values were normalized to total cell numbers in the culture vessels at each time point. To determine the main mode of cell death in PPS-exposed cultures, an ApoTox-Glo™ Triplex Assay (Promega) was performed. Thereby, the effects on viability, cytotoxicity, and apoptosis induction were assessed simultaneously at 24 hours and 7 days after exposure. The assay was performed according to manufacturer’s instructions.

### Western blot analysis for PARP-1

In order to discriminate between apoptotic and necrotic cell death, a Western blot for poly (ADP-ribose) polymerase (PARP-1) was performed 7 days after exposing microcarrier cultures to both, PPS and CNTs. To obtain a positive control, cells were treated with 1 µg/ml staurosporine for 5 hours. Upon washing cells with ice-cold PBS, cell lysates were prepared in RIPA buffer (50 mM Tris-HCl pH 8.0, 150 mM NaCl, 1% Triton X-100, 0.5% sodium deoxycholate, 0.1% SDS; Sigma) overnight at 4 °C with the addition of protease and phosphatase inhibitor cocktails (1 tablet each was dissolved in 10 ml RIPA buffer) (Roche Diagnostics, Austria). The protein concentration was determined photometrically by a Bradford Assay (Bio-Rad Laboratories, California). 20 µg of the protein lysate was separated by SDS-PAGE (NuPage 4–12% Bis-Tris gels; Life Technologies, Austria) and transferred to nitrocellulose membrane (Bio-Rad). Following primary antibodies were used: PARP (dilution: 1∶750; Cell Signaling Technology, Massachusetts), and as a loading control beta-Actin (diluted 1∶1000; Sigma). As secondary antibody we used a goat anti-rabbit (Cell Signaling Technologies) or rabbit anti-mouse HRP-conjugated antibody, respectively (DAKO, Denmark) at a final concentration of 1 µg/ml. An overnight incubation at 4 °C was performed for both primary antibodies, followed by incubation with secondary antibodies at room temperature for 2 hours. Specific protein bands were visualized by the enhanced chemiluminescence assay (Amersham Biosciences, Germany).

### Statistical analysis

All experiments, except long-term exposure to PPS, were repeated three times. At least triplicates were assessed for each sample. Due to the small sample size (n = 3), all data are described descriptively, as mean ± standard deviation (SD). To investigate the effects of PPS on cell growth over time (n = 5), repeated measurements ANOVA was performed. For post-hoc analysis, a Bonferroni correction was conducted. A p-value <0.05 was considered to indicate statistical significance. The statistical analysis was performed by using the statistical software SPSS Version 20.

## Results

Prior to the assessment of the long-term effects in 3D culture, NPs were characterized according to their physicochemical parameters and to their acute cytotoxic action in conventional culture.

### Particle characterization

The hydrodynamic sizes of PPS were larger when diluted in medium, especially in the presence of FBS than indicated by the supplier. The differences between the indicated size and the hydrodynamic radius in medium with 10% FBS, were more pronounced for the 20 nm PPS than for the 200 nm PPS. All particles were negatively charged except 200 nm PPS when dissolved in DMEM with 10% FBS. Sizes of the >50 nm CNTs increased markedly when protein-free medium was used. A summary of the particle properties is presented in Table 1.

**Table 1:** Characterization of tested NMs.

Particle	Size (nm)			ζ-pot.(mV)		
	Aqua bidest.	DMEM	DMEM+10% FBS	Aqua bidest.	DMEM	DMEM+10% FBS
PPS 20 nm	22.76	28.89	73.59*/12.37	−37.1	−13.4	−11.7
PPS 200 nm	211.6	211.5	224.8	−57.6	−11.0	8.08
Fluoro-Max 20 nm	22.22	44.56	58.26	−11.1	−28.2	−10.3
MWCNT >50 nm	n.d.^‡^	602.9	50.41	n.d.^‡^	−16.9	−11.0

* Predominant peak is indicated first, ^‡^Not determined due to aggregates.

### Acute cytotoxicity

**Figure 1:**
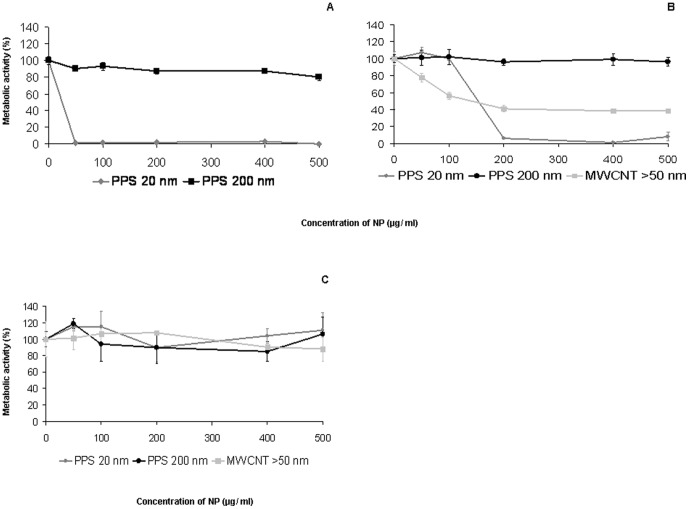
Acute cytotoxicity of NPs exerted on EAhy 926 in different cell cultures after 24 hours. Cells in conventional cultures were treated with NPs dissolved in serum-free medium (A) as well as in medium with 10% FBS (B). Cells cultured on microcarriers were exposed to NMs dissolved in medium with 10% FBS. Data are presented as mean ± SD.

After exposure of cells to 20 nm PPS for 24 hours, the cell viability was reduced in a dose-dependent manner (Fig. 1 A). Addition of serum to the medium decreased the cytotoxicity of the particles. The estimated IC_50_ was approximately 120 µg/ml in DMEM supplemented with 10% FBS and 30 µg/ml in medium without FBS. The viability of cells after exposure to 20 nm PPS did not decrease when cells were grown on GEM^TM^. 200 nm PPS exhibited no cytotoxicity, irrespective of the medium used (Fig. 1 A–C). Exposure of cells to CNT dissolved in serum-containing DMEM decreased cell viability similarly to 20 nm PPS (Fig. 1 B); the estimated IC_50_ was approximately 125 µg/ml. No changes in cell viability were detected when cells were seeded onto GEM™ (Fig. 1 C).

### Mode of action

**Figure 2:**
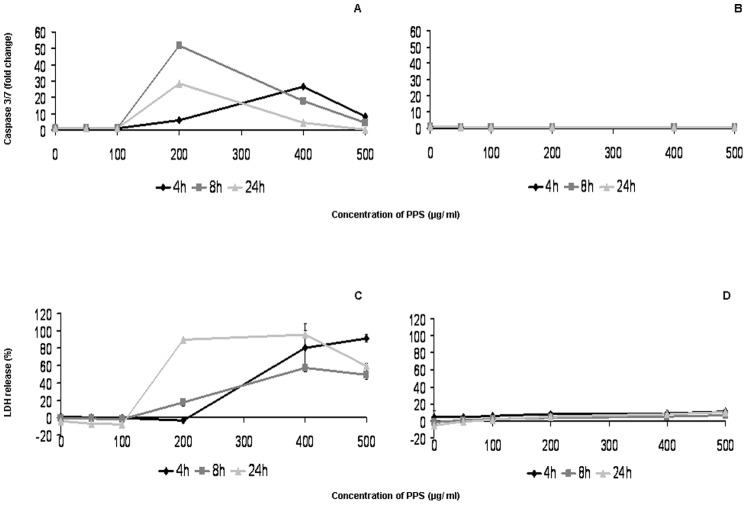
Mode of action of PPS in conventional cultures. Activation of caspases 3 and 7 (A and B) and release of LDH (C and D) upon exposure of EAhy 926 cells to 20 and 200 nm PPS for 4, 8, and 24 hours compared to untreated cells. Data are presented as mean ± SD. (h), hours.

Low concentrations (<200 µg/ml) of 20 nm PPS had no influence on membrane integrity or on activation of caspases 3 and 7 at any time-point. However, PPS at 200 µg/ml induced strong activation already after 4 hours, reaching the maximum of an about 50-fold increase after 8 hours and an almost 30-fold increase after 24 hours as compared to non-exposed cells (Fig. 2 A). A similar dose-dependent effect was observed for membrane disruption (Fig. 2 C). Exposure of the cells to 200 nm PPS resulted in a very low release of LDH (up to 15% of the lysis control) and no notable increase in caspase activation at any time-point (Fig. 2 B and D).

### Establishment of the microcarrier cell culture system

**Figure 3:**
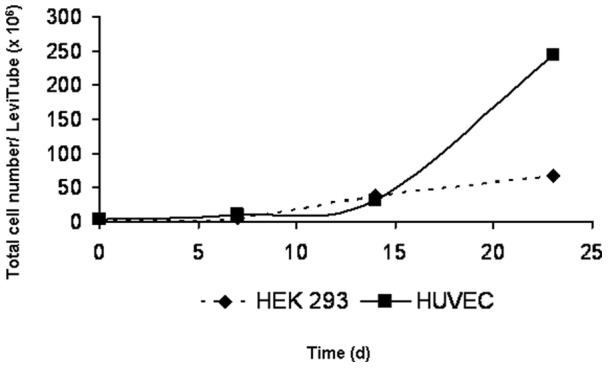
Growth curve of EAhy 926 cultured on basal membrane coated GEM™. Two pre-installed protocols for cell culturing epithelial (HEK 293) and endothelial (HUVEC) cells were compared. (d), days.

**Figure 4:**
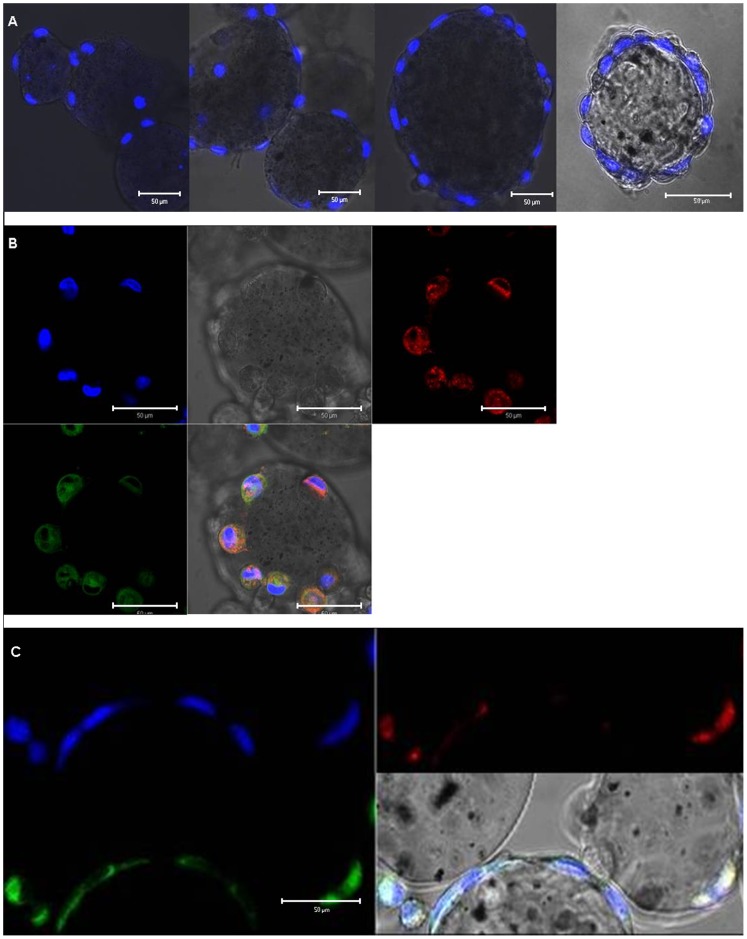
EAhy 926 attached to GEM™. Nuclear staining with 5 µg/ml Hoechst 33342 was performed 1, 5, 7, and 14 days after inoculation (A). Vital dye staining for ER and mitochondria five days after inoculation. Hoechst 33342 dye was used as nuclear counterstain (B). Internalized red fluorescent NPs co-localize with the lysosomal dye LysoSensor™ Green DND-189 but not with the nucleus (blue) (C).

For optimization of the exposure system, different coatings of the GEM™ (gelatine, laminin, fibronectin, collagen type I and IV, basal membrane) as well as different incubation protocols, pre-defined by the supplier, were compared. Cells that were seeded on basal membrane coated microcarriers and cultured according to the protocol for endothelial cells (HUVEC) reached approximately 4 times higher cell densities compared to those when the protocol for epithelial cells (HEK 293) was used (Fig. 3). Cultures were maintained for 23 days without sub-culturing. The doubling time of EAhy cells was lower in the microcarrier culture system (70 h) than in conventional cultures (30 h). Staining with Hoechst 33342 dye showed an increase in cell proliferation on GEM™ (Fig. 4 A). Furthermore, vital staining for mitochondria and endoplasmic reticulum (ER) demonstrated the viability of the cells (Fig. 4 B).

### Intracellular localization of PPS

Cells were exposed to 20 µg/ml of the red fluorescent PPS in order to study cellular localization. R25 were observed within the cells, mainly localized in lysosomes (Fig. 4 C).

### Long-term effects of NPs in microcarrier culture

**Figure 5:**
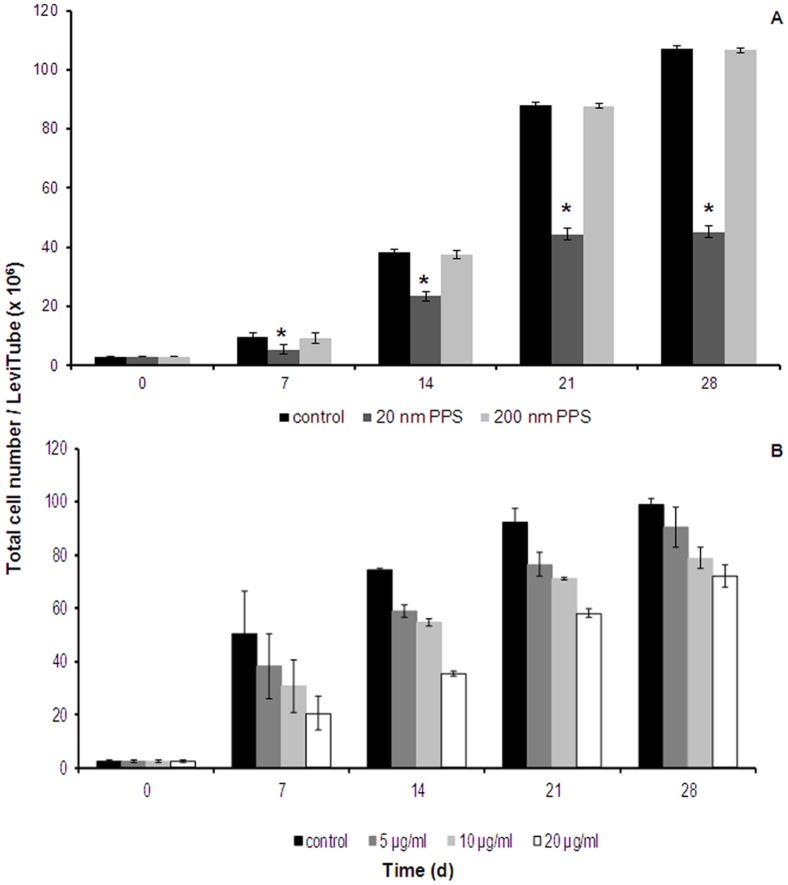
Long-term cytotoxic effects of NPs on EAhy 926. Cultures were exposed to PPS over a period of 28 days, Data are presented as mean ± SD of the total cell number; n = 5, *p*-value <0.05. * indicates statistically significant changes in cell numbers between control and treated cells at each time-point. Long-term effects upon exposure to different concentrations of MWCNT >50 nm are shown in (B). Data are presented as mean ± SD of the total cell number per culture vessel; n = 3. (d), days.

Cells were cultured according to the established protocol (basal membrane coated GEM^TM^, and incubation protocol for endothelial cells) for four weeks with a medium change performed once per week. After inoculation, NPs were added at a concentration 250 times lower than the concentration where cytotoxicity was seen in the acute cytotoxicity setting (24 hours). Exposure of the cells to 20 µg/ml of 20 nm PPS resulted in a significantly reduced cell number already after 7 days, showing a decrease in cell numbers of approximately 50%. Even stronger effects were observed at later time-points. No decrease in cell number was observed when the cells were exposed to 20 µg/ml of 200 nm PPS (Fig. 5 A).

Exposure to concentrations of 5–20 µg/ml of CNT decreased cell numbers in a dose-dependent manner at day 7 to approximately 75%–40% of control cells, respectively. However, with prolonged contact the cell populations recovered. The recovery rate was more rapid for cells exposed to 20 µg/ml than for cells that were exposed to 5 µg/ml and the values reached 90%–70% of the control after 4 weeks (Fig. 5 B). Cell viability was not impaired at any time-point.

### Mode of action in microcarrier cultures

**Figure 6:**
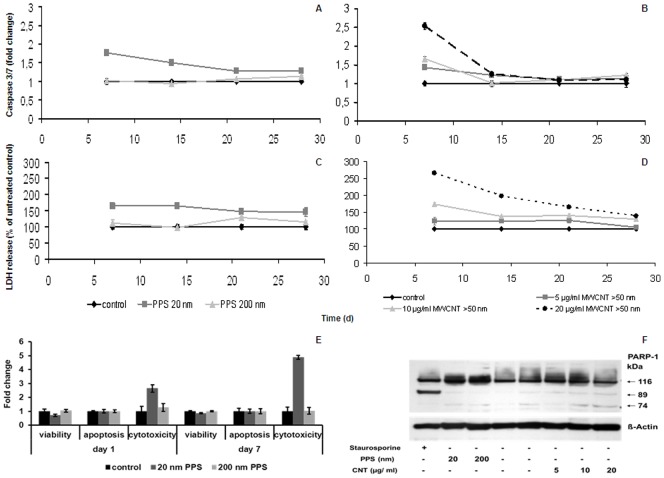
Mode of action of different NPs in microcarrier cultures. Induction of apoptosis (A and B) and necrosis (C and D) after long-term exposure of EAhy 926 grown on GEM™ to NPs. Data are presented as mean ± SD, normalized to the total cell numbers per culture vessel; (d), days. Changes in viability, caspase activation, and cytotoxicity in cells exposed to PPS at early time-points are presented in (E). Data are presented as mean ± SD. Western blot detecting PARP-1 after treatment of microcarrier cultures with both, PPS and CNTs at an early time-point (day 7) is presented in (F). Treatment with 1 µg/ml staurosporine was used as a control for apoptosis induction.

Long-term exposure to 20 nm PPS induced an 80% higher activation of the effector caspases 3 and 7 after 7 days as compared to the control cells. Over time, the induction of apoptosis decreased to about 30% of the untreated control (Fig. 6 A). 20 nm PPS induced necrosis with a maximum of LDH release, about 65% higher than in control cells, after 7 days of culturing (Fig. 6 C). At later time-points, LDH release slightly dropped by about 15%. However, exposure to 20 nm PPS resulted in a 2.5- to 5-fold increase in cytotoxicity, while the viability was slightly reduced, as detected by the ApoTox-Glo™ Triplex Assay. No induction of apoptosis was detected (Fig. 6 E). Exposure to 200 nm PPS induced neither apoptosis nor necrosis at any time-point (Fig. 6 A, C, E). CNT induced both, apoptosis and necrosis, in a dose-dependent manner, reaching a 2.5-fold increase as compared to the control upon exposure to a concentration of 20 µg/ml (Fig. 6 B and D). Similar to PPS, the strongest induction of caspases and the highest release of LDH occurred after 7 days of exposure, after which the levels approached those of control cells.

### Western blot for PARP-1

As shown in Fig. 6 F, an 89 kDa fragment representing cleaved PARP-1, indicative for apoptotic cell death, appeared exclusively in the staurosporine treated positive control. Treatment with NPs provoked the appearance of not only un-cleaved (116 kDa), but also additional slight cleaved PARP-1 bands (89 kDa, 72 kDa), which, in combination, occur only after necrosis.

## Discussion

In this study, the long-term cytotoxicity testing with the BioLevitator™ was used for the identification of potential chronic effects of NPs on cells and was compared to conventional cell culture. This system enabled the culture of viable cells to high densities and identified adverse cellular effects of 20 nm PPS and of >50 nm CNTs.

A microcarrier cell culture system enables cell culturing at higher cell densities for a longer time period. The polarized cell growth, in a more physiologic environment than in conventional cell culture vessels, allows a better differentiation of the cells [37]. The mimicked *in vivo* situation slows down proliferation and therefore the nutrients are depleted more slowly. In addition, due to their structure, the porous microcarrier facilitates long-term cell culturing as the nutrients from the medium and the molecules secreted from the cells (e.g. growth factors) are retained inside the beads. Due to the small volume of medium required to feed the cells over the entire culturing period compared to conventional cell culture methods, it is a very cost- and material-saving method. Bioreactors were initially developed to increase the yield of cellular products (e.g. antibodies) [38]. This culture may also be suitable for toxicity testing and/or the identification of long-term effects. This is particularly important for NPs because they have been shown to persist in organisms [39], and are influenced by several factors, such as medium composition, binding of proteins, mechanical pre-treatment, and pH, which makes it very laborious to evaluate all these parameters *in vivo*. The GEM™ technology and the BioLevitator™ allowed the culture of viable cells with high reproducibility. As expected, the physiological growth on basal membrane coated microcarriers slowed down the proliferation of EAhy 926 cells, which is advantageous for the study of NP accumulation. One potential limitation of the system is that high-throughput testing is not possible because only four experiments can be performed in parallel.

The endothelial cells EAhy 926 can be exposed to relatively high concentrations (100 µg/ml) of 20 nm PPS for 24 hours without showing any apparent damage in a conventional culture. In microcarrier culture, the resistance to the toxic action of PPS is even higher. Two mechanisms could be suggested that may explain the lower cytotoxicity of PPS in microcarrier culture: a more physiological growth with a better supply of nutrients and the fact that a smaller area of the cell membrane is accessible to the PPS because cells are more densely packed [40]. Upon longer incubation times, however, the situation is inversed. The low concentrations of the NPs did not have a strong influence on the proliferation of cells maintained in conventional cell culture, but pronounced cytotoxicity was detected in microcarrier culture. This difference may be caused by a higher dilution of the intracellular concentration of NPs due to proliferation. Our experiments proved that the doubling rate of EAhy 926 cells in conventional culture was 2.3 times higher than in the microcarrier culture. At concentrations higher than 100 µg/ml, 20 nm PPS decreased the metabolic activity of the cells in conventional culture and induced the activation of caspases 3 and 7. In addition, an increased release of LDH, as an indicator of membrane damage, was also observed after exposure to these doses of PPS for 24 hours. Particles of 200 nm did not exert any effect upon culturing under the same conditions. Upon acute exposure, the main modes of PPS induced cell death were found to be apoptosis and necrosis. Fröhlich *et al*. investigated the impacts of 20 nm carboxylated polystyrene (CPS) NPs in the same cell line, grown in conventional cell culture for 24 hours, and also demonstrated induction of necrosis and apoptosis [41]. This similarity between 20 nm CPS and 20 nm PPS may be linked to their similar physicochemical parameters: the differences in size (42 nm (CPS) vs. 73 nm (PPS)) were small and the surface charge of both particles was slightly negative. Upon prolonged exposure to PPS, not only LDH release was increased as compared to controls, but also the activation of caspases. However, it is very unlikely that both modes of cell death are induced at the same time. The contradictory findings on caspase activation (Fig. 6 A) could be explained by the normalization of very small differences in assay values (caspase 3/7) between untreated and treated cells versus larger differences in total cell numbers of the respective culture. Moreover, all other data supported the induction of necrosis as the predominant mode of action of 20 nm PPS upon long-term exposure. Collectively, we detected no induction of apoptosis and only low induction of necrosis at each time-point in cells exposed to 20 µg/ml of 20 nm PPS. As both cell death mechanisms should occur within 24 hours [42], we presume that the reduction in cell number observed upon long-term exposure was also caused by the decreased cell proliferation in the BioLevitator^TM^, as the lower doubling rate of the cells in microcarrier cultures promotes the accumulation of NPs.

The BioLevitator™ bioreactor used in this study, also appears suitable for the assessment of biological effects upon exposure to other NMs. CNTs could find broad medical application, particularly in imaging and treatment (vaccination, hyperthermia) provided they are not overtly toxic [21]. Long-term exposure in the microcarrier culture showed a dose-dependent decrease in cell numbers after 7 days. With prolonged contact the cell populations recovered. These findings were supported by our data on the mode of action since the peak levels of induction of apoptosis and/or necrosis were also detected at day 7. At later time-points, activation of caspases or a notable release of LDH was not detected. The BioLevitator^v^bioreactor may also be used for the toxicological assessment of conventional compounds. The action of drugs on cytochrome P450 (CYP) enzymes is important for the metabolization by hepatocytes. Testing is complicated by the fact that CYP enzyme activities are low or absent not only in hepatocyte cell lines but also in cultured primary hepatocytes [43]. In preliminary experiments on HepG2 cells growing on microcarriers, we observed high cell density and a higher activity of the enzyme CYP1A1, important for many pathways (e.g. steroid hormone biosynthesis, tryptophan metabolism, retinol metabolism, metabolism of xenobiotics, and metabolic pathways) (data not shown). Findings on HepG2 cells grown in a three dimensional cell culture and the advantage of that culturing method were described in many other studies [44], [45]. Long-term culture in the BioLevitator™ may therefore also be suitable to evaluate certain aspects of metabolization by hepatocytes.

In summary, our findings suggest that non-biodegradable NPs persist in cells and may cause cell damage. Due to the localization of the NPs in lysosomes, as supported by our data on fluorescent labelled particles, it is necessary to investigate their effect on lysosomes. Lysosomes are potential targets for drug-induced damage, such as for drug-induced lysosomal phospholipidosis resulting in lysosomal dys-function [46].
